# Digital teaching tools in sports medicine: A randomized control trial comparing the effectiveness of virtual seminar and virtual fishbowl teaching method in medical students

**DOI:** 10.1371/journal.pone.0267144

**Published:** 2022-06-16

**Authors:** Stefan Hertling, Doreen Hertling, Georg Matziolis, Ekkehard Schleußner, Franziska Loos, Isabel Graul

**Affiliations:** 1 Department of Gynecology and Obstetrics, University Hospital Jena, Jena, Germany; 2 Orthopaedic Department, Campus Eisenberg, University Hospital Jena, Jena, Eisenberg, Germany; 3 Fakultät für Gesundheit (Department für Humanmedizin), Lehrstuhl für Medizintheorie, Integrative und Anthroposophische Medizin, Witten/Herdecke, Germany; 4 Department of Obstetrics, University Hospital Jena, Jena, Germany; 5 Department of Gynecology, Hospital Rummelsberg, Schwarzenbruck, Germany; 6 Practice for Orthopaedics and Shoulder Surgery, Leipzig, Germany; 7 Department of Trauma, Hand and Reconstructive Surgery, University Hospital Jena, Jena, Germany; 8 Department für Orthopädie, Unfall - Universitätsklinikum Halle, Halle, Germany; Imam Abdulrahman Bin Faisal University, SAUDI ARABIA

## Abstract

**Background:**

Since the COVID-19 pandemic, the demand for online courses has increased enormously. Therefore, finding new methods to improve medical education is imperative.

**Objective:**

The aim of this study was to compare the self-reports of the individual student-centered virtual teaching techniques (seminar versus fishbowl) in a group of medical students.

**Methods:**

During the second semester of 2020, students in the clinical phase of the study (n = 144) participated in the optional subject of Sports Medicine. The students were divided into 2 groups. One group (n = 72) received the knowledge transfer in the form of a virtual seminar, the other group (n = 72) in the form of a virtual fishbowl.

**Results:**

Virtual seminar and virtual fishbowl students gave insights into these teaching techniques. Most of the students from the virtual fishbowl group believed that the virtual fishbowl format allowed them to be more actively involved in learning. The mean quiz scores were statistically higher for students in the virtual fishbowl group than students in the virtual seminar group (p < 0. 001).

**Conclusion:**

This study concluded that virtual seminars and virtual fishbowl formats could be served as structured learning and teaching formats. At the same time, the virtual fishbowl format can promote an active exchange of knowledge from students’ perspectives.

## Introduction

COVID-19 has caused unprecedented disruption to the medical education process and to healthcare systems worldwide [1]. Medical faculties worldwide have faced the challenge of adapting instruction to digital platforms–classroom lectures, seminars and clinical placements can no longer be performed in the usual face-to-face settings [2]. To maintain the quality of medical training under the circumstances of the COVID-19 pandemic, medical educators need to think outside the box [3]. As a solution, digital technologies have been used to support innovative teaching on e-learning platforms, virtual training, or videoconferencing [4, 5]. Therefore, the teaching methods of the medical education curriculum must evolve to provide students with opportunities for continuous learning without delay due to the pandemic. Although the pandemic of COVID-19 appeared as an unusual catalyst for promoting e-learning, it is still unclear whether students are offered sufficient online teachings in terms of quantity and quality to complete their medical education successfully [6]. The search for new methods to improve medical education is imperative. Since the COVID-19 pandemic, virtual lectures have been the backbone of many universities’ education. Although this method is controversial, it is by far the most efficient method of delivering complex subjects with many contents to a large group of students, introducing new and difficult subjects, and providing comprehensive overviews and summaries. This form of lecture does not necessarily lead students to understand the content. An alternative to the classical lecture is active teaching. Students take an active part in this process. The focus is shifted away from the teacher to the students (student-centered approach) and allows them to actively acquire knowledge through meaningful activities and reflection [7]. This is intended to enable students to solve higher-level cognitive tasks such as problem-solving, critical thinking and reflection, instead of memorizing them. Different forms of active education have been proposed for medical education [2]. The Fishbowl method is a teaching method for dynamic group participation. The basic structure consists of two concentric circles (groups), alternating between discussion and observation groups. The students discuss a relevant topic or case in the inner circle while the observation group surrounds them. Students in the outer circle silently observe the discussion, identify themes and patterns, and assess the validity and merits of the inner group’s arguments. The main objective is to familiarize students with the structure and characteristics of an in-depth interactive discussion as a learning tool. Accordingly, this format can serve as a problem-solving or decision-making aid to generate divergent views, promote team building and improve communication between groups [8]. The aim of the study was to compare students’ perceptions of the effectiveness of two virtual student-centered teaching methods, the virtual seminar, and the virtual fishbowl methods, in two groups of medical students.

## Methods

The study protocol was reviewed and approved by the University of Jena, Faculty of Medicine Scientific Ethical Committee (reference number 2019-1456-Bef). The curriculum of the Faculty of Medicine at the University of Jena comprises a six-year, results-oriented program that spans 12 semesters. The first four semesters are part of preclinical training. Upon completing the first stage examination, students will undergo clinical training from the fifth semester onwards. The clinical training lasts a total of six semesters and ends with the second stage examination (5th till 10th semester). After that, the practical year takes place in the 11th and 12th semesters. Upon completion of the third stage examination, the medical degree is obtained. Subsequently, the students can undergo further training in different specialties. In the clinical training phase, students must take optional elective subjects and earn 80 points with these. Various optional elective subjects are available. The elective subject of sports medicine was launched in the first semester of 2019 and has since established itself successfully. This elective subject is intended to teach a variety of disciplines from sports medicine. In addition to the essential basic theoretical topics in sports medicine, practical teaching contents such as examination techniques, preparation of training plans and ECG evaluations are also being taught. With the elective sports medicine subject, 28 points can be obtained after completing the subject. The content of the course is conveyed using practical case studies from everyday clinical practice. The duration of the course is eight weeks. Once a week there is a lesson of 90 minutes duration. Students choose their electives via the university’s internal digital learning platform. The number of recruitments for the elective sports medicine subject is limited to 36 students. For the second semester of 2020, the number increased to 72 students to form another study cohort. Thus, there were two sports medicine courses in the second semester of 2020. Both courses had to be held digitally due to the COVID-19 pandemic. Students were randomly assigned to the virtual seminar group or the virtual fishbowl group. Baseline adaptive randomization with the biased coin method was used for this study [9, 10].

Both courses are taught by three lecturers. Although those lecturers came from different disciplines, all have additional recognized qualification and credential in teaching sports medicine. In Germany, the state medical chamber awarded the additional designation of sports medicine after completing further curriculum study and passing the final exam. The three lecturers are also trained in ’medical didactics’ and have the essential skill of digital teaching. A course coordinator gave final feedback, and the session ended with a quiz. The student is considered to have completed the elective course once the student passes the case presentation and quiz. A certificate for course completion was then issued.

### Objective and hypothesis

This study aimed to determine any significant difference in the students’ active participation between the virtual seminar (classical method) with the virtual fishbowl method. The hypothesis is that the students in the virtual fishbowl group participate in the class more actively.

### Sample size

**Fig 1:**
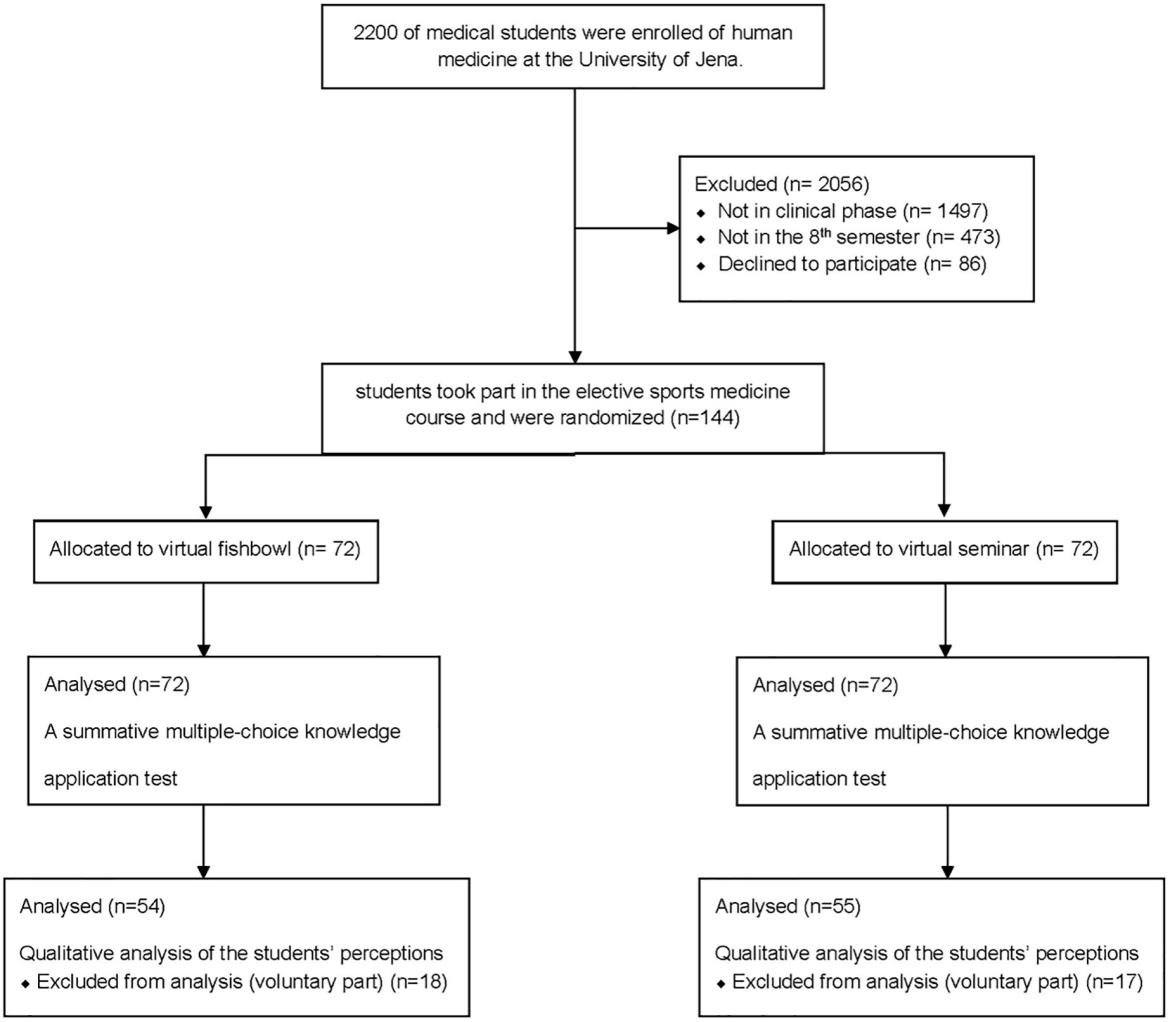
Schematic flow chart of the students’ enrollment process.

A total of 2,200 medical students were enrolled at the University of Jena in the academic year 2020/ 2021. Of these, 703 students were studied in the clinical phase. Out of the 703 students, 230 were in the 8th semester. The sample size was calculated based on the population size of 230, the confidence level of 95%, the margin of error of 5%, so an ideal sample size was 144 students. Of these 230 students, 144 students in the second academic year 2020 took the elective sports medicine course. The enrollment process of the study is shown in Fig 1.

### Virtual seminar implementation

**Fig 2:**
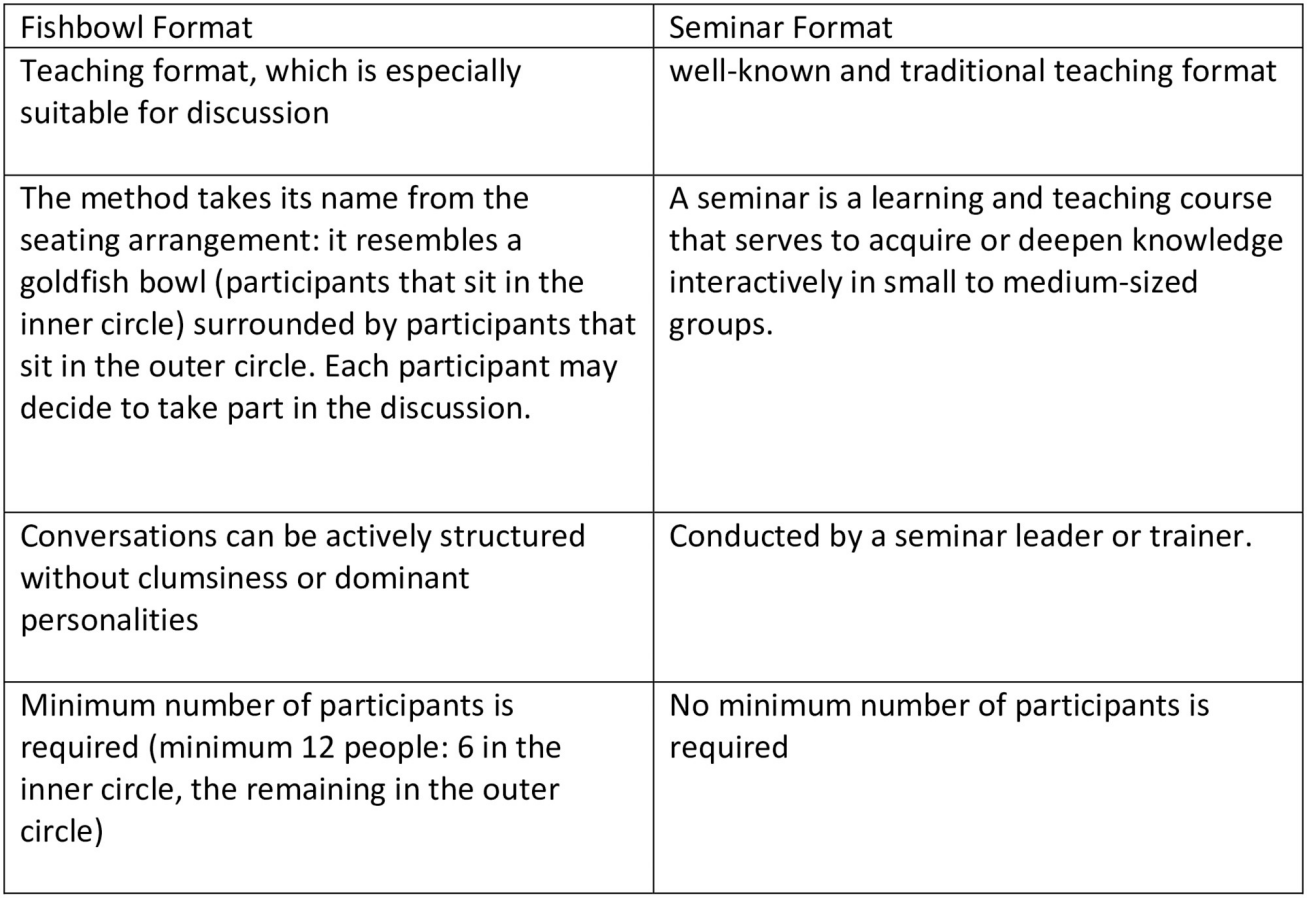
The important differences between the fishbowl and seminar format.

The students in the virtual seminar group (control group) attended mandatory weekly seminars. After that, the students were divided into 6 teams, with each team consisting of 12 students. Each team received a real sports medicine clinical case with the medical history, relevant photos, and results of laboratory tests and/ or X-rays about one week before the oral case presentation. Students were expected to study as a group and to present the case to the rest of the class via a 15-minute PowerPoint^®^ presentation. This was followed by a 20-minute discussion in which the teaching staff monitored each session. The lecturers encouraged discussion among the students during and after the presentations while occasionally intervening with challenging questions. Each team presented twice during the semester and received two different clinical cases with different pathologies. Anecdotal evidence from previous courses indicated that not all students actively participated during the peer presentations; some students were passive and ignored the oral presentations. The seminars (started in the first semester of 2019) incorporated some of the methods proposed in the literature to improve [11] the interaction between the presenting team of students and their classmates to enhance the overall commitment in the learning process (Fig 2). These methods included brainstorming, think-pair sharing, and buzz group sessions before, during and after the presentations. The seminar’s contents were presented virtually via the Zoom^®^ online telecommunication platform. At the end of each seminar session, the course coordinator discussed the learning experience and provided feedback on the clinical cases presented. A summative multiple-choice questions knowledge application quiz (a total of 12 marks) was conducted for all students. The presenting students received extra marks for their two presentations during the course using a known rubric, with each student adding 14 seminar marks.

### Virtual fishbowl method

By switching to digital teaching methods in the academic year of 2020, we have decided to try something new. In the second semester (July to December) of 2020, we used the virtual fishbowl training method for the second group of students. As in previous academic years, the learning objectives of the elective sports medicine subject remained unchanged. After initial planning and training of the lecturers during the holiday, a member of the faculty of the elective sports medicine subject led a workshop for all enrolled students on the functioning of the virtual fishbowl training method, including a demonstration on a mock case (typical injuries in professional sports versus grassroots sports). This training aimed to give students an understanding of the new method and practice its dynamics by conducting a series of discussions to facilitate reflection, explain and solve problems. All workshop materials were uploaded to the e-learning platform so that students could work through them at their own pace. Like the virtual seminars, one real clinical case was uploaded to the e-platform weekly (12 cases in total). In contrast to the seminar arrangement, a more structured case format was provided. Each clinical case included 20 open questions on pathogenesis, diagnosis, and treatment planning, focusing on depth of understanding, to be prepared by all students. Based on Bloom’s taxonomy [12], the rationale for this format was that all students should come to the virtual fishbowl session with a general understanding of the case that would be presented. Later, all students had to deal with the clinical scenario by applying and analyzing what they had learned and coming out with a diagnosis and treatment plan. There were two subgroups for each virtual fishbowl session at the mandatory weekly virtual fishbowl sessions (participants were controlled): the discussion and observation group. Each group of students in the last case presentation started in the inner circle (the discussion group), while the students in the next case presentation sat in the outer circle (the observation group). Afterward, the inner group (the fishbowl) discussed and tried to solve the sports medicine issues of the assigned case together for 30 minutes, while the group of students sitting in the outer circle watched and listened to the conversation and took notes. In the following 10 minutes, the observation subgroup took part in the discussion, correcting and asking questions to the fishbowl (discussion) subgroup to supplement the sports medicine content of the case.

Next, both subgroups changed roles and followed the same protocol. This means that the students who were now in the inner circle spent 30 minutes trying to solve the problems of the same case by coming out with a diagnosis and treatment plan, while the students sat in the outer circle watched in silence. Like the previous setting, the students in the inner circle were interviewed by those in the outer circle for 10 minutes. In the virtual fishbowl session, 6 students formed the inner circle, and 12 students formed the outer circle. The number of students was evenly distributed. One faculty member per group supervised and encouraged discussions, led inquiries, and justified the general understanding of the case. Notes were taken in a well-known column to assess the depth of each student’s intervention and understanding. Finally, after each lesson, all small groups of ’breakout’ rooms were brought back to the Zoom^®^ main room for a large group session. As in the seminars, the course coordinator discussed the learning experience and gave feedback of the clinical case. Finally, all students underwent a summative multiple-choice questions knowledge application quiz. Each student got 14 fishbowl marks.

### Student’s perception questionnaire

Before completing the elective sports medicine subject, all students were asked to complete an anonymous perception e-questionnaire to determine how well they have dealt with the course formats. The questionnaire consisted of ten items on a five-point Likert scale [13] and a final open-ended question that allowed free-text answers asking for "comments for improvement." The perceptions’ questionnaire was conducted by members of the Working Group Young Forum of the German Society for orthopedics and trauma surgery (Arbeitsgemeinschaft Junges Forum der Deutschen Gesellschaft für Orthopädie und Unfallchirurgie (DGOU)). A panel of experts conducted a questionnaire in two separate online meetings to investigate the identified areas of interest based on individual literature searches, like the EULAR-recommended standard operating procedures [14]. The study questionnaires have a web-based design according to published guidelines for questionnaire research [15–17]. The choice of questions for the questionnaire was based on both comparable work and the quality criteria for questionnaires [18]. Members of the Working Group Young Forum of the German Society for orthopedics and trauma surgery (DGOU) were asked to provide feedback on the validation process’s format, completeness, clarity, and procedure [15, 17]. The survey was pilot tested. The survey was administered to 10 students to gauge the need to refine wording and format and to check whether the predefined response options were exhaustive. Minor revisions were made accordingly. Finally, a 10-items, self-administered questionnaire was developed. It consisted of questions in categorical Likert scales (5 levels) and one open question and was entitled ’Sports Medicine’.

### Data analysis

The marks for the multiple-choice questions knowledge application quiz for both virtual fishbowl and virtual seminar methods were evaluated. The students’ subjective perception of both digital teaching methods was analyzed descriptively. Subsequently, several student’s t-tests were used to compare the results of the seminar questionnaire with those of the fishbowl. Only fully completed questionnaires were included in the analysis. The free-text answers "comments to improve", which were provided by students for both seminar and fishbowl group, were analyzed according to the principles of content analysis. All written comments were grouped (by one researcher) by topic, using an "open coding" method to list the data analytically [13]; this was to be ensured that all aspects of each theme were considered [19]. Subsequently, the comments were compared, and the conceptually similar comments were identified and grouped according to topics. The mean values of the quiz potencies of students who completed seminar and fishbowl sessions in the same academic year were calculated. The reliability of the weekly quizzes was determined with the help of Cronbach’s alpha [20]. The students’ t-tests were used to compare the results of seminars and fishbowl group. The statistical analyses were conducted using Microsoft Excel 365 (Windows Inc. Florida, USA) and the IBM SPSS Statistics for Windows version 27.0 (IBM Corp. Armonk, NY, USA). The significant level was set at the level of p < 0.05.

## Results

Of the 144 students who successfully completed the elective sports medicine subject, 72 were assigned to the virtual seminar group, and the remaining were assigned to the virtual Fishbowl group. The mean age of students in both groups was 25 years (the virtual fishbowl group was 25.07 ± 1.97 years, the virtual seminar group was 24.95 ± 2.42 years). Of the virtual seminar group students, 62 (86.0%) completed the perception questionnaire and 55 (76.0%) provided qualitative comments. For virtual fishbowl group students, 61 (85.0%) completed the perception questionnaire and 54 (72.0%) provided qualitative comments. As the students’ perception questionnaire was optional and shall not be compulsory for all the students who took the elective sports medicine subject according to the university regulation, not all of them filled out the questionnaire. The subject’s study framework did not emphasize the teaching-learning process of the lecturers and students, as evidenced by the digital evaluation of the recorded lectures of both groups. The average active speaking time of students in the virtual seminar group was 35.53 minutes per 90-minute unit, while the average active speaking time of students in the virtual fishbowl group was 48.21 minutes.

### Quantitative analysis of the students’ perceptions

Table 1 shows the average responses to the perception questionnaire for both groups. Students in the virtual seminar group rated question 2: "How true is it that the course allowed me to be actively involved?" with the lowest average score of 3.1 ± 1.02. In contrast, the virtual fishbowl group rated question 10: "How true is it that the course that I would recommend this digital teaching format to fellow students?" with the highest average score of 4.4 ± 0.6. In addition, five out of the ten questions were rated significantly better by the students of the virtual fishbowl group as compared to the virtual seminar group, which were question 1: "How true is it that the course allowed me to learn?", question 2: "How true is it that the course allowed me to be actively involved?", question 6: "How true is it that the course met my expectations?", question 8: "How true is it that the course made it possible to convey practical relevance?" and question 9: "How true is it that the course made it possible to communicate case reports actively?". The remaining questions did not show any statistically significant difference.

**Table 1:** Mean score (SD) for student’s perception questionnaire from both groups (1 = Strongly disagree, 2 = disagree, 3 = neutral, 4 = agree, 5 = Strongly agree). Student’s t-test results compared the seminar questionnaire results with those from the fishbowl.

Question	How true is it that the course	Virtual Seminar group	Virtual fishbowl group	p value
		(n = 72)	(n = 72)	
1	… allowed me to learn?	3.3 (1.1)	3.9 (0.6)	**p < 0.001**
2	… allowed me to be actively involved?	3.1 (0.7)	4.1 (0.9)	**p < 0.001**
3	… was delivered using an attractive format?	3.5 (0.6)	3.8 (1.1)	p = 0.4994
4	… met the set learning outcomes?	3.6 (1.2)	3.9 (0.6)	p = 0.0598
5	… assessment was fair and acceptable?	3.7 (0.9)	3.8 (0.5)	p = 0.4112
6	… met my expectations.	3.2 (0.7)	3.9 (1.0)	**p < 0.001**
7	… included important subject for my training as a doctor?	3.9 (0.4)	4.0 (0.5)	p = 0.1872
8	… made it possible to convey practical relevance?	3.2 (1.1)	4.2 (0.4)	**p < 0.001**
9	… made it possible to communicate case reports actively?	3.4 (0.8)	3.9 (1.2)	**p = 0.0038**
10	… that I would recommend this digital teaching format to fellow students?	4.2 (0.6)	4.4 (0.9)	p = 0.1189

### Qualitative analysis of the students’ perceptions

**Fig 3:**
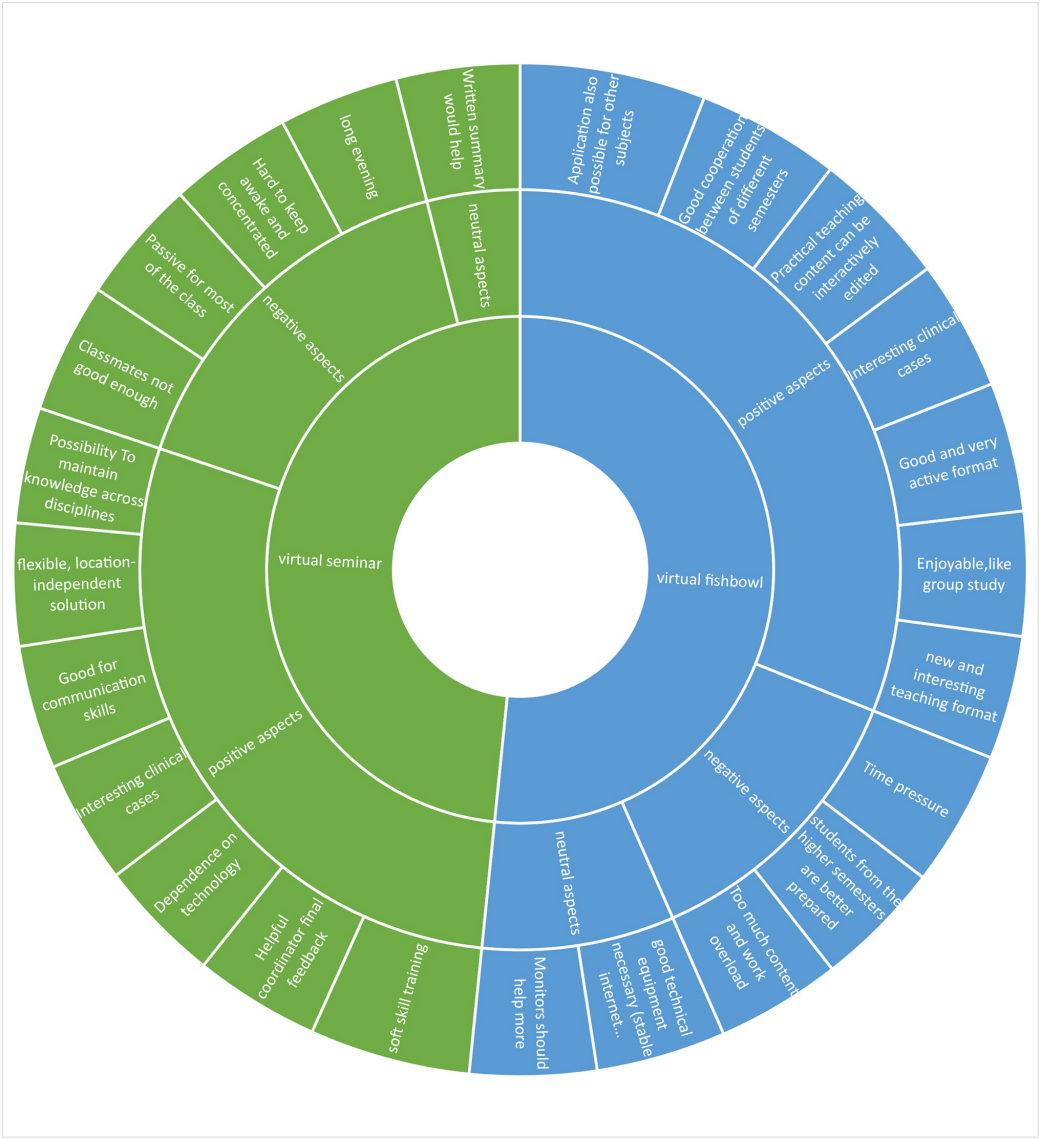
Suggestions or comments for improvement from the students’ perceptions.

The number of students from the virtual seminar or virtual fishbowl group that provided comments or suggestions for improvement after completing the elective sports medicine subject was 55 and 54, respectively. Comments or suggestions for improvement were divided into 12 thematic areas as shown in Fig 3.

### Summative quiz and presentation scores

The students of both groups took the same quiz at the end of the session. The mean scores of the seminar quiz and the presentation (n = 6) were 78.1% (range 58.2%–84.9%) for the students from the virtual seminar group and 81.4% (range 65.0%–88.1%) for the students of the virtual fishbowl group, and the alpha coefficient of Cronbach was 0. 681 for the virtual seminar group and for the virtual fishbowl format Cronbach’s alpha coefficient was 0. 773. There was a statistically significant difference in mean scores between the two groups (p < 0.001). The group of the virtual fishbowl format was significantly better than the group of the virtual seminar.

## Discussion

The study investigated how the medical students perceive the effectiveness between virtual seminar and virtual Fishbowl teaching methods as the digital teaching tool for elective sports medicine subject. Virtual seminars or virtual fishbowl method was used to stimulate discussion between the students who had prepared a clinical case earlier and presented it to the rest of the class later. In these training methods, the learning process developed by exchanging opinions between the students who prepared the clinical case earlier and presented their work to the other students later [21]. In comparison, the average active speaking time of the students in the virtual seminar method was shorter. This result can be attributed to the differences in our students’ quantitatively measured learning approaches. Students from the virtual seminar group showed similar learning approaches, while students from the virtual fishbowl group showed a higher level of active participation during the class. This result partly explains why more students in the virtual fishbowl group preferred the virtual seminar method, as it seems to create a higher intrinsic motivation in the virtual fishbowl method. Conversely, more students in the virtual seminar group may need to be explained what to do and how to do it, as they seem to have less intrinsic motivation to learn. Therefore, students may require a low-threshold, digitally structured, student-centered teaching technique such as the virtual fishbowl method. As highlighted in the literature, the students’ non-participation is a negative aspect of active group work [22]. In the digital education age, this negative aspect has gained importance. Many teachers complain about this point in virtual courses [23]. Most students in the virtual seminar group took the role of a passive listener. Factors such as group size, students’ interest, and the virtual seminar duration play important roles [24]. A large group may have also hindered the active participation of students. In addition, it was found that few students gained a real understanding of the clinical cases that they had not personally prepared for the presentation. Although this study showed mixed results, the fundamental aim of testing the virtual fishbowl method was to search for an effective alternative virtual group teaching method that could stimulate and encourage the active participation of the entire class. This particular pedagogical approach of the virtual fishbowl was chosen for this study because it allows and promotes participation through give-and-take experiences without threatening discussions, while at the same time offering the opportunity to learn from peers and solve problems together in the virtual form [22]. The theoretical basis of our study was Kolb’s experimental learning theory. The acquisition of content is based on the interaction of content and experience, whereby both transform each other and can thus be used digitally in the delivery of practical teaching content [25]. Some of the topics from the students’ comments on the virtual seminar and fishbowl method (Fig 3) correspond to the literature [26], which should include an active learning experience: cooperation between peers (fishbowl), fun (fishbowl), teacher as moderator (seminar and fishbowl) and improvement of communication skills (seminar). However, both methods had common themes: "interesting clinical cases" and "helpful coordinator at the end of feedback". Practical teaching content can be interactively edited according to the literature [27]. These comments can be interpreted as "motivating, relevant and useful feedback from the moderator" and can be seen as essential for the success of active virtual group lessons. A 14-week study of 38 students to promote peer collaboration found that the fishbowl technique "solves certain research problems and provides preserved advice" [28]. Our students’ comments from the virtual fishbowl group are like those of other German students who took the political education course [29].

The fishbowl method was found to be more “funny or interesting” than the plenary method of student teaching, although the class was conducted virtual instead of live. In a further study, 128 students from the psychology department were given an understanding of interpretation using the fishbowl method and three other methods [30]. Self-efficacy improved after using all formats, but the students found the fishbowl method the least helpful. However, the authors explained that most students only "observed" the fishbowl discussion for time reasons. This previous study suggested that the fishbowl method should only be used when everyone could participate, which tally with our study results.

The students of the virtual fishbowl method admitted that they felt time pressure and were overloaded with information because too much content was shared within the limited time frame. Therefore, when using the virtual fishbowl format, clear and well-defined content should be outlined to avoid these negative aspects [31, 32]. The reason for these results is not clear from the present study, but it is likely that students from the virtual fishbowl group tend to think that the less structured, more flexible seminar format is more oriented towards the specific clinical problem solving, for which even the passive students can engage better. Alternatively, as the literature suggests, the wide range of mixed skills, including soft skills and different students’ educational backgrounds may also explain why our students preferred the higher level of organization and structure of the virtual fishbowl method [33]. The results of this study cannot be easily generalized. Although the sample size was sufficient for reliable analysis, we report the results of a single cohort of mixed students in virtual classroom formats with only two student-centered training methods. Many other techniques to facilitate structured discussions have not yet been sufficiently evaluated [34]. For the teaching of the dental students, situational learning, patient-doctor role-playing games and storytelling proved to be other useful methods, which were used in addition to the clinical training phase of prospective dentists. The training was carried out according to a European educational profile, the Association for Dental Education (ADE) profile [35]. The ADE profile includes the competencies of future European dentists. This should help the dental faculties in Europe to further harmonize and improve the quality of their curricula. Such virtual teaching methods with defined educational content are currently lacking for the education of medical students in Germany and the whole of Europe.

Potential biases may have been introduced into the study design because only the eighth-semester students were recruited to compare two teaching methods in this study. A cross-sectional study with students from other semesters would have avoided this bias. Another factor to consider when comparing virtual seminar and virtual fishbowl digital teaching method is the cost. At our facility, introducing the fishbowl method required more staff than the virtual seminar, which increased the cost of each course. The virtual seminar was conducted exclusively by the course coordinator, while the virtual fishbowl method required two additional tutors who had to be trained intensively before the teaching started [13].

## Conclusion

Most medical students stated that the new virtual fishbowl method allowed them to participate more actively and learn more effectively than the virtual seminar method. In contrast, the students felt that there was less time pressure, less workload and not overloaded with too much content in the virtual seminar compared to the virtual fishbowl method. Therefore, we believed we could improve the virtual seminar session for our students by trying an innovative and student-centered learning approach, which is the virtual fishbowl method, which is especially useful in the delivery of active practical content. The results of this study and our study experience potentially could help other faculty members who want to try a different method to optimize the pedagogical value of their virtual group learning, such as the virtual fishbowl method.

## Supporting information

S1 File (PDF)
