# Supplementary material for: Digital teaching tools in sports medicine: A randomized control trial comparing the effectiveness of virtual seminar and virtual fishbowl teaching method in medical students
Source: PLoS One. 2022 Jun 16;17(6):e0267144. doi: 10.1371/journal.pone.0267144 (PMC9202876; doi:10.1371/journal.pone.0267144)
Supplement: S1 File — (PDF) [file pone.0267144.s001.pdf]

student number	virtual fishbowl	virtual seminar	Q1	Q2	Q3
1	1	0	4	5	3
2	1	0	3	5	4
3	1	0	5	3	3
4	1	0	5	4	4
5	1	0	5	5	3
6	1	0	5	3	3
7	1	0	4	2	3
8	1	0	3	4	4
9	1	0	5	5	4
10	1	0	5	4	4
11	1	0	4	3	3
12	1	0	5	4	4
13	1	0	4	5	3
14	1	0	4	4	4
15	1	0	4	3	3
16	1	0	5	4	4
17	1	0	4	4	3
18	1	0	3	5	4
19	1	0	4	4	4
20	1	0	5	4	4
21	1	0	4	5	5
22	1	0	3	5	5
23	1	0	4	4	5
24	1	0	5	3	3
25	1	0	5	4	4
26	1	0	4	5	5
27	1	0	5	4	4
28	1	0	4	5	3
29	1	0	3	4	4
30	1	0	4	4	5
31	1	0	3	5	5
32	1	0	4	4	4
33	1	0	5	5	5
34	1	0	4	4	4
35	1	0	3	4	4
36	1	0	3	3	5
37	1	0	4	4	4
38	1	0	5	5	5
39	1	0	4	4	5
40	1	0	3	4	4
41	1	0	4	3	4
42	1	0	3	3	3
43	1	0	4	4	4
44	1	0	5	5	5
45	1	0	4	4	4
46	1	0	3	3	4
47	1	0	3	4	4

48	1	0	3	5	4
49	1	0	4	4	3
50	1	0	3	4	3
51	1	0	4	5	4
52	1	0	5	4	3
53	1	0	4	3	4
54	1	0	3	5	5
55	1	0	4	4	4
56	1	0	3	4	3
57	1	0	3	5	4
58	1	0	5	4	3
59	1	0	4	4	4
60	1	0	3	5	4
61	1	0	4	4	3
62	1	0	3	4	3
63	1	0	3	3	3
64	1	0	4	3	3
65	1	0	3	4	4
66	1	0	5	4	3
67	1	0	4	4	3
68	1	0	3	5	2
69	1	0	4	4	4
70	1	0	5	5	3
71	1	0	4	4	4
72	1	0	3	4	3
73	0	1	3	3	3
74	0	1	3	3	3
75	0	1	4	3	3
76	0	1	4	3	3
77	0	1	4	4	4
78	0	1	5	3	3
79	0	1	3	3	3
80	0	1	3	3	3
81	0	1	4	4	4
82	0	1	3	3	3
83	0	1	4	2	5
84	0	1	3	3	3
85	0	1	3	4	4
86	0	1	3	3	3
87	0	1	4	4	4
88	0	1	5	3	4
89	0	1	5	4	4
90	0	1	4	4	4
91	0	1	3	3	3
92	0	1	3	3	4
93	0	1	4	4	4
94	0	1	4	4	4
95	0	1	3	4	4

96	0	1	5	4	4
97	0	1	4	3	4
98	0	1	3	3	3
99	0	1	4	4	4
100	0	1	3	3	3
101	0	1	3	3	3
102	0	1	4	3	3
103	0	1	3	3	3
104	0	1	3	3	3
105	0	1	3	3	3
106	0	1	3	4	4
107	0	1	3	3	4
108	0	1	3	3	3
109	0	1	3	3	4
110	0	1	5	4	3
111	0	1	3	3	4
112	0	1	3	4	4
113	0	1	3	3	4
114	0	1	4	4	3
115	0	1	3	3	3
116	0	1	2	3	4
117	0	1	3	3	4
118	0	1	4	4	4
119	0	1	2	2	5
120	0	1	4	2	4
121	0	1	3	3	3
122	0	1	2	2	4
123	0	1	3	3	4
124	0	1	4	3	3
125	0	1	3	3	5
126	0	1	2	2	4
127	0	1	3	3	4
128	0	1	2	4	3
129	0	1	2	2	2
130	0	1	2	5	3
131	0	1	3	3	3
132	0	1	4	3	5
133	0	1	3	3	3
134	0	1	3	3	4
135	0	1	3	3	3
136	0	1	4	2	4
137	0	1	3	2	4
138	0	1	3	3	3
139	0	1	3	3	2
140	0	1	4	3	3
141	0	1	3	3	4
142	0	1	4	2	2
143	0	1	3	3	2

144	0	1	3	3	3

Q4	Q5	Q6	Q7	Q8	Q9	Q10
3	5	4	5	5	4	5
4	4	3	4	4	4	4
3	3	5	3	5	3	5
4	4	4	3	5	3	5
3	4	4	4	4	4	4
3	3	4	5	4	5	4
3	4	3	4	3	4	4
4	4	5	4	4	4	4
4	3	4	5	3	5	5
4	4	3	4	4	4	4
3	4	4	3	5	3	5
4	4	4	3	4	3	4
3	2	3	5	5	5	5
4	4	5	4	4	4	4
3	4	3	4	4	4	4
4	4	3	4	5	4	5
3	5	3	3	3	3	4
4	4	4	5	5	5	5
4	3	5	4	4	4	4
4	3	4	4	4	4	5
5	5	3	4	4	4	4
5	4	4	5	5	5	5
5	5	4	4	4	4	4
3	3	3	3	5	3	5
4	4	4	4	4	4	4
5	5	5	5	5	5	5
4	3	3	4	4	4	4
3	3	4	5	4	5	4
4	3	5	4	4	4	4
5	5	4	4	5	4	5
5	4	3	5	5	5	5
4	4	4	4	4	4	4
5	5	5	5	5	5	5
4	4	4	4	4	4	4
4	4	4	4	4	4	4
5	5	5	5	5	3	5
4	4	4	4	4	4	4
5	5	5	4	3	4	4
5	5	5	5	4	3	4
4	4	4	4	5	4	5
4	4	3	4	3	3	5
3	3	4	3	3	4	4
4	4	4	4	4	4	4
5	5	5	5	5	4	5
4	4	4	3	4	3	4
4	4	3	5	3	4	5
4	4	4	4	4	4	4

5	4	5	5	5	4	5
3	3	4	3	4	3	4
3	3	4	4	4	4	4
4	4	3	5	5	3	4
4	3	4	4	4	4	4
4	4	4	4	3	4	4
5	5	5	3	5	4	4
4	4	4	4	4	4	4
3	3	3	3	4	4	5
4	4	4	5	5	3	5
3	3	3	4	4	4	5
4	4	4	4	4	3	4
5	4	5	4	5	4	5
3	3	3	4	4	4	5
4	3	4	3	4	5	4
3	3	3	4	3	3	3
5	3	5	4	3	4	4
4	4	4	3	4	3	4
3	3	3	3	4	4	5
4	3	4	3	4	4	4
2	2	2	4	5	4	4
4	4	4	4	4	3	5
3	3	3	4	5	5	5
4	4	4	4	4	5	5
4	3	4	4	4	4	4
3	5	3	4	3	3	5
4	4	3	3	3	3	4
3	5	3	3	3	3	5
4	4	3	4	3	3	5
4	4	4	5	4	4	4
3	4	3	4	3	3	4
3	4	3	4	3	3	4
3	5	3	5	3	3	4
4	4	4	4	4	4	5
3	4	3	3	3	3	4
5	3	2	3	5	5	5
3	4	3	5	3	3	4
4	3	4	4	4	4	5
3	3	3	4	3	3	4
4	3	4	4	4	4	4
4	4	3	3	4	4	5
4	5	4	5	4	4	4
4	4	4	4	4	4	5
3	3	3	4	3	3	3
4	4	3	4	4	4	5
4	3	4	5	4	4	4
4	3	4	4	4	4	3
4	4	4	3	4	4	4

4	5	4	4	4	4	2
4	3	3	5	4	4	3
3	2	3	4	3	3	5
4	4	4	5	4	4	4
3	5	3	4	3	3	4
3	3	3	4	3	3	4
3	3	3	5	3	3	5
3	3	3	4	3	3	5
4	4	3	5	3	3	4
3	3	3	4	3	3	5
5	5	4	4	4	4	4
4	4	3	3	3	3	4
4	3	3	4	3	3	5
5	4	3	4	3	3	4
4	5	3	3	4	4	3
3	3	3	4	3	4	4
4	4	4	3	4	3	5
4	4	3	4	3	3	3
5	4	3	4	4	3	4
3	3	3	4	3	3	4
4	5	4	3	3	4	4
5	5	3	4	3	3	4
4	3	4	4	4	4	5
4	4	3	4	2	3	4
3	3	4	3	2	3	4
3	4	3	4	3	3	4
3	3	3	3	2	3	4
5	4	3	4	3	5	4
3	3	4	4	3	3	4
4	4	3	4	3	5	4
4	4	3	4	2	4	4
3	4	3	4	3	2	4
3	5	3	3	4	3	5
3	3	3	4	2	4	5
3	3	3	3	4	5	5
3	4	3	4	3	3	4
3	3	3	4	3	4	5
3	5	3	5	3	3	5
3	3	3	3	3	4	4
4	4	3	4	3	4	3
4	4	3	3	3	3	4
3	3	3	4	2	3	4
3	3	3	4	3	4	5
3	4	3	4	3	3	4
3	3	4	3	3	3	4
4	3	3	5	3	3	5
5	3	3	5	3	3	5
4	3	3	4	3	3	5

	3	3	3	5	3	3	4

student number	virtual fishbowl	virtual seminar	age
1	1	0	24
2	1	0	26
3	1	0	28
4	1	0	24
5	1	0	25
6	1	0	27
7	1	0	22
8	1	0	28
9	1	0	24
10	1	0	26
11	1	0	25
12	1	0	22
13	1	0	25
14	1	0	25
15	1	0	30
16	1	0	22
17	1	0	23
18	1	0	24
19	1	0	27
20	1	0	23
21	1	0	25
22	1	0	25
23	1	0	26
24	1	0	21
25	1	0	24
26	1	0	25
27	1	0	24
28	1	0	24
29	1	0	24
30	1	0	32
31	1	0	26
32	1	0	31
33	1	0	24
34	1	0	25
35	1	0	25
36	1	0	26
37	1	0	26
38	1	0	25
39	1	0	27
40	1	0	22
41	1	0	26
42	1	0	24
43	1	0	27
44	1	0	25
45	1	0	28
46	1	0	22
47	1	0	24

48	1	0	27
49	1	0	26
50	1	0	24
51	1	0	22
52	1	0	23
53	1	0	26
54	1	0	26
55	1	0	27
56	1	0	22
57	1	0	25
58	1	0	25
59	1	0	26
60	1	0	25
61	1	0	30
62	1	0	24
63	1	0	23
64	1	0	22
65	1	0	29
66	1	0	25
67	1	0	29
68	1	0	25
69	1	0	25
70	1	0	24
71	1	0	23
72	1	0	21
73	0	1	23
74	0	1	26
75	0	1	25
76	0	1	24
77	0	1	25
78	0	1	27
79	0	1	22
80	0	1	28
81	0	1	24
82	0	1	26
83	0	1	26
84	0	1	24
85	0	1	25
86	0	1	25
87	0	1	32
88	0	1	21
89	0	1	23
90	0	1	24
91	0	1	27
92	0	1	23
93	0	1	25
94	0	1	25
95	0	1	26

96	0	1	23
97	0	1	24
98	0	1	26
99	0	1	24
100	0	1	24
101	0	1	24
102	0	1	30
103	0	1	28
104	0	1	31
105	0	1	24
106	0	1	25
107	0	1	26
108	0	1	27
109	0	1	24
110	0	1	25
111	0	1	26
112	0	1	21
113	0	1	26
114	0	1	27
115	0	1	23
116	0	1	25
117	0	1	26
118	0	1	26
119	0	1	27
120	0	1	25
121	0	1	23
122	0	1	23
123	0	1	22
124	0	1	23
125	0	1	26
126	0	1	26
127	0	1	27
128	0	1	22
129	0	1	24
130	0	1	25
131	0	1	26
132	0	1	25
133	0	1	24
134	0	1	24
135	0	1	26
136	0	1	22
137	0	1	26
138	0	1	25
139	0	1	26
140	0	1	23
141	0	1	25
142	0	1	21
143	0	1	23

	144	0	1	25

questionare completed	qualitative comments completed
1	1
1	1
1	1
1	1
1	1
1	1
1	1
1	1
1	1
1	1
1	1
1	1
1	1
1	1
1	1
1	1
1	1
1	1
1	1
1	1
1	1
1	1
1	1
1	1
1	1
1	1
1	1
1	1
1	1
1	1
1	1
1	1
1	1
1	1
1	1
1	1
1	1
1	1
1	1
1	1
1	1
1	1
1	1
1	1
1	1
1	1
1	1

	1	1
	1	1
	1	1
	1	1
	1	1
	1	1
	1	1
	1	0
	1	0
	1	0
	1	0
	1	0
	1	0
	1	0
	0	0
	0	0
	0	0
	0	0
	0	0
	0	0
	0	0
	0	0
	0	0
	0	0
	0	0
	1	1
	1	1
	1	1
	1	1
	1	1
	1	1
	1	1
	1	1
	1	1
	1	1
	1	1
	1	1
	1	1
	1	1
	1	1
	1	1
	1	1
	1	1
	1	1
	1	1
	1	1
	1	1
	1	1

1	1
1	1
1	1
1	1
1	1
1	1
1	1
1	1
1	1
1	1
1	1
1	1
1	1
1	1
1	1
1	1
1	1
1	1
1	1
1	1
1	1
1	1
1	1
1	1
1	1
1	1
1	1
1	1
1	1
1	1
1	0
1	0
1	0
1	0
1	0
1	0
1	0
1	0
0	0
0	0
0	0
0	0
0	0
0	0
0	0
0	0
0	0
0	0

0	0

mean values of quiz-score and presentation
65
88
88
85
84
87
87
69
86
84
84
86
78
82
87
77
85
82
83
88
88
80
77
81
78
82
79
87
76
78
65
77
86
67
79
88
68
70
81
73
78
79
88
82
80
85
84

81
82
84
78
76
88
79
85
78
81
79
74
84
80
82
78
79
81
82
80
87
84
82
88
88
86
84
77
84
58
84
82
84
82
83
82
79
84
75
84
77
78
84
77
82
81
78
83

78
81
83
81
79
84
80
82
81
76
78
65
77
79
82
79
78
78
70
71
73
78
79
82
82
80
84
74
82
79
65
78
76
78
74
75
78
79
84
82
84
76
79
78
79
81
78
76

seminarformat	average speaking time in minutes per 90 minutes unit
virtual fishbowl	48.21
virtual seminar	33.53
